# Assessment of Urinary Stone Chemical Compositions and Prevalence of Metabolic Disorders Among Urolithiasis Patients in Northern Sri Lanka: A Prospective Study

**DOI:** 10.7759/cureus.63377

**Published:** 2024-06-28

**Authors:** Balagobi Balasingam, Sriskantharajah Varothayan, Sittampalam Rajendra, Vinojan Satchithanantham, S T Sarma, Sujeenthan Sri Pandurangana, Sunthareswaran Vithyasahar, Thirunavukkarasu Jothini, Srivadivel Vishnuja, Shathana Paramanathan

**Affiliations:** 1 Urology, Jaffna Teaching Hospital, Jaffna, LKA; 2 Interventional Radiology, Jaffna Teaching Hospital, Jaffna, LKA; 3 Surgery, Faculty of Medicine, University of Jaffna, Jaffna, LKA; 4 Physiology, Faculty of Medicine, University of Jaffna, Jaffna, LKA

**Keywords:** metabolic evaluation, urolithiasis, sri lanka, calcium oxalate, analysis

## Abstract

Introduction

Urolithiasis, a common urological disorder affecting the global population, demonstrates geographical diversity due to factors such as water quality, climate variations, health conditions, and dietary habits. This study, conducted in Northern Sri Lanka, examines urinary stone compositions and assesses the prevalence of metabolic disorders among urolithiasis patients.

Methods

This prospective cross-sectional study, conducted at Jaffna Teaching Hospital, Jaffna, Sri Lanka, from July 2022 to June 2023, focused on surgically treated urolithiasis patients. Institutional ethical clearance was obtained. Patient details and investigational findings were collected through questionnaires and data extraction forms. Stone analysis utilized Fourier transform infrared spectroscopy, and a detailed metabolic evaluation of a 24-hour urine collection sample was carried out.

Results

This study followed 153 surgically treated urolithiasis patients, primarily male (64.3%), with a mean age of 48.64. Ureteric colic (48.4%) was common, with kidney stones (45.8%) prevalent; 57.52% had recurrent stones. Diabetes mellitus (DM; 23.5%) was the top comorbidity. Calcium oxalate monohydrate (COM) stones (78.4%) were the most frequent, followed by uric acid (12.4%). COM predominated in the 40-59 age group. There was no significant gender-stone type association. A total of 86.9% had metabolic abnormalities, notably hypocitraturia (60.1%). Moreover, 23% had both hypocitraturia and hypomagnesuria. Some metabolic disorders showed gender differences, with a marginal age-metabolic disorder association (p < 0.061). Urine oxalate levels were normal, with higher variability in males.

Conclusion

Middle-aged males with urolithiasis commonly presented with ureteric colic and predominantly had COM stones. Recurrent stones were common, often accompanied by metabolic abnormalities such as hypocitraturia and hypomagnesuria, with DM as the primary comorbidity.

## Introduction

Urolithiasis ranks among the most common global urological conditions, with a prevalence estimated at 8-13% [1]. The incidence varies regionally due to differences in drinking water quality, dietary patterns, climate, obesity rates, and other health conditions [1].

A diet that lacks variety and heavily relies on rice, along with the quality of the water and the hot climate, are factors contributing to this high occurrence. These conditions are also prevalent in Sri Lanka, making it likely to be a part of the stone belt [2,3].

The region’s dry zone climate leads to frequent dehydration, concentrating minerals in urine, and promoting stone formation. Hard water, high in calcium and magnesium, along with potential contaminants, further exacerbates this risk. The predominantly vegetarian diet, rich in oxalates from foods like spinach and nuts, combined with high rice consumption, affects urinary pH and stone composition. Socioeconomic factors, such as occupations involving heavy physical labor in high temperatures and limited access to healthcare, contribute to higher stone formation risks. Genetic predispositions and cultural practices also play roles in stone prevalence. Understanding these factors is crucial for identifying risk factors, tailoring management options, and implementing effective preventive approaches specific to the region.

Regions with hard water, high in calcium and magnesium, are more prone to calcium-based stones, while contaminants like heavy metals can also affect stone composition. Hot and humid climates increase dehydration, concentrating urine and promoting stone formation, especially uric acid (UA) stones. Seasonal changes further impact hydration and dietary habits, influencing stone composition by increasing urine concentration during hotter periods. Understanding these factors is essential for effective treatment, prevention, and prognostication of urinary stones [3,4].

Previous studies on nephrolithiasis are retrospective and have predominantly focused on analyzing the chemical composition of renal stones, often with limited sample sizes [5,6]. However, a significant gap exists in the literature regarding the comprehensive analysis of metabolic abnormalities associated with urolithiasis. While some studies have touched upon metabolic factors including hypercalciuria, hyperoxaluria, hyperuricosuria, hypocitraturia, hypomagnesuria, hyperphosphaturia, and urinary pH variations, they have not been thoroughly investigated alongside stone composition analysis. Hence, there is a need for research that integrates both aspects to provide a more holistic understanding of nephrolithiasis etiology and management.

This study aims to assess the diverse chemical compositions of urinary stones in Northern Sri Lanka and determine the prevalence of metabolic disorders, including hypercalciuria, hyperoxaluria, hyperuricosuria, hypocitraturia, hypomagnesuria, hyperphosphaturia, and variations in urinary pH, among urolithiasis patients seeking treatment at the region’s sole tertiary treatment center. By integrating stone composition analysis with the evaluation of metabolic abnormalities, the study seeks to provide comprehensive insights into the etiology and management of nephrolithiasis in this specific geographical context.

## Materials and methods

This is a prospective, laboratory-based, cross-sectional investigational study conducted among patients with urolithiasis who underwent surgical interventions at Jaffna Teaching Hospital, Jaffna, Sri Lanka, from July 2022 to June 2023. The study was designed to gather comprehensive data on the clinical and biochemical profiles of these patients, contributing valuable insights into the epidemiology and pathophysiology of urolithiasis in the region.

Prior to the initiation of the study, ethical clearance was obtained from the ethics review committee of the institution, as indicated by the approval reference number J/ERC/22/133/NDR/270. Informed written consent was also obtained from all participants, ensuring that they were fully aware of the study’s aims and procedures and agreed to contribute their data voluntarily.

The data collection was meticulously structured using two primary tools. Firstly, an interviewer-administered questionnaire was employed to gather detailed patient information. This included demographic data such as age and gender, clinical presentation details such as the nature of their complaints, as well as family history and comorbid conditions that might influence the occurrence of urolithiasis. The use of an interviewer-administered approach ensured the accuracy and completeness of the data collected, as participants were guided through the questionnaire by trained personnel.

Secondly, a dedicated data extraction form was used to systematically record the investigational findings. This included results from a variety of diagnostic tools and tests: ultrasound scans of the kidneys, ureters, and bladder (USS/KUB), non-contrast computer tomography (NCCT/KUB), and detailed stone analysis. Biochemical analyses of serum samples were conducted to measure levels of calcium, phosphate, UA, and creatinine. Urinalysis was also performed, which included a full urine report and a 24-hour urine analysis to quantify the excretion levels of calcium, oxalate, citrate, UA, magnesium, and phosphate. These tests provided a comprehensive overview of both the metabolic and anatomical factors contributing to stone formation in the patients.

The chemical composition of the kidney stones was analyzed using Fourier transform infrared spectroscopy, a sophisticated technique that allows for precise identification of the mineral components of the stones. The classification of these stones was based on the system proposed by Abdel-Halim et al., which offers a standardized method for categorizing stone types based on their chemical composition [7]. This aspect of the study is crucial for understanding the etiology of stone formation and tailoring appropriate treatment and preventive strategies for patients with different types of urolithiasis.

## Results

A total of 153 surgically treated urolithiasis patients were recruited over a year for the study, and the majority were men (64.3%). The mean age of the patients was 48.64 (SD = 14.7). The majority of patients fell into the 40-59 age group (46.4%) (Table 1).

**Table 1:** Demographic and clinical characteristics

Characteristics		n	%
Sex	Male	98	64.3
	Female	55	35.7
Age in years	<20	6	3.9
	20-39	36	23.5
	40-59	71	46.4
	60-79	40	26.1
Occupation	Students	3	2
	Skill workers	93	60.8
	Non-skill workers	57	37.2
Presenting complaints	Ureteric colic	74	48.4
	Loin pain	25	16.3
	Abdominal pain (nonspecific)	14	9.2
	Lower urinary tract symptoms	20	13.1
	Hematuria	3	2
	Asymptomatic	6	3.9
Diagnosis	Renal stone	69	45.8
	Stag horn calculi	17	11.1
	Non-stag horn calculi	52	34
	Ureteric stone	67	43.8
	Bladder/urethral stone	16	10.5
Recurrent stone	Yes	88	57.5
	No	65	42.5

Diabetes mellitus (DM, 23.5%) was the most common comorbidity associated with urolithiasis patients, followed by hypertension and hyperlipidemia. Nearly a quarter of patients (26.1%) had high serum creatinine at the time of the surgical procedure, and in the majority of them, renal function improved during follow-up.

Ureteric colic was by far the most common complaint, with 48.4% of the patients presenting with it, followed by loin pain (16.3%) and lower urinary tract symptoms (13.1%). A smaller proportion presented with nonspecific abdominal pain or hematuria. Six (3.9%) patients were detected incidentally (Table 1).

The most prevalent stone location was the kidney, with 45.8% of patients having renal stones. Ureteric stones were also a common diagnosis in 43.8% of patients, while bladder and urethral stones were less common (10.5%). Notably, the majority of patients (57.52%) had recurrent stones.

**Figure 1:**
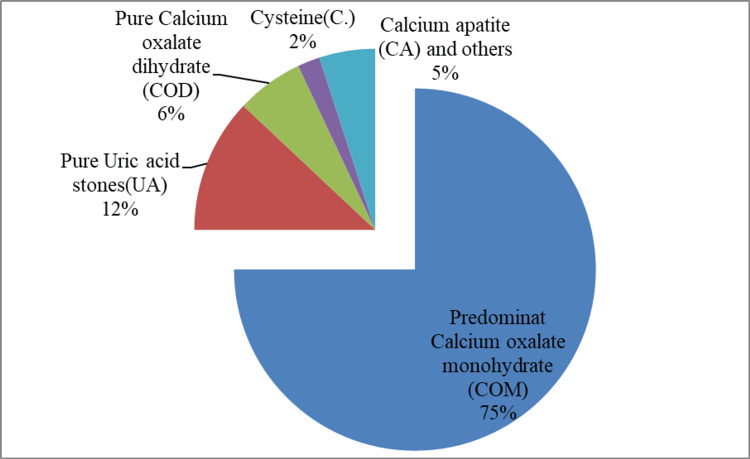
Chemical composition of stones

The distribution of the types of stones based on chemical composition is illustrated in Figure 1. The majority of the stones were calcium oxalate monohydrate (COM)-predominant stones, accounting for more than three-quarters of the cases (78.4%). Pure UA stones amounted to 12.4% of the cases. Pure calcium oxalate dihydrate (COD), cysteine, and calcium apatite (CA) stones were found in the remaining cases.

Overall, the dataset indicates a male predominance in urolithiasis cases, with males having a higher prevalence across all stone types compared to females. However, there was no statistically significant association between the type of stone and gender (p = 0.917).

The most prevalent stone type is COM across all age groups, with the highest count in the 40-59 age group (n = 61), followed by UA stones, which showed an increasing trend with age, peaking in the 60-79 age group with eight cases. All cases of CA belonged to the 40-59 age group. Overall, the dataset suggested that the prevalence of different stone types varied across age groups. There was no significant association between the type of stones and the age groups of patients (p = 0.222).

The mean urine oxalate levels were within the normal range for both male and female cohorts. However, the variability, as indicated by the SD and IQR, was higher in males than in females. The mean urine calcium level was below the midpoint of the normal range, while the urine mean phosphorus level was below the lower end. The mean urine UA level was within the normal range and was relatively consistent, as indicated by the smaller IQR compared to the mean. Overall, these indicated most components of urinary metabolic profiles were within the normal range, with some components nearing the limits of the normal range in individuals (Table 2). A small percentage of the patients (13.1%) had no metabolic abnormality, and the majority (86.9%) had at least a single metabolic abnormality (Table 3).

**Table 2:** Metabolic parameters measured in a 24-hour urine analysis

Parameters		Mean ± SD	IQR
Urine oxalate	Male (0.08-0.49 mmol/24 hours)	0.31 ± 0.48	0.2
	Female (0.04-0.32 mmol/24 hours)	0.21 ± 0.17	0.16
Urine calcium (100-300 mg/24 hours)		152.89± 98.19	132.795
Urine phosphorus (500-750mg/24 hours)		443.46 ± 205.18	273.43
Urine citrate (288-902 mg/24 hours)		272.11 ± 149.93	176.075
Urine magnesium (60-210 mg/24 hours)		70.61 ± 32.00	27.37

**Table 3:** Prevalence of metabolic abnormalities

Metabolic abnormality	Male (n)	Female (n)	Total, n (%)
Hyperoxaluria	11	11	22 (14.4)
Hypercalciuria	7	2	9 (5.9)
Hyperuricosuria	8	2	10 (6.5)
Hypocitraturia	60	32	92 (60.1)
Hypomagnesuria	27	27	54 (35.3)
Hyperphosphaturia	10	7	17 (11.1)

Hypocitraturia is the most prevalent metabolic abnormality in the population (60.1%), followed by hypomagnesuria (35.3%) and hyperoxaluria (14.4%). Hyperphosphaturia (11.1%), hyperuricosuria (6.5%), and hypercalciuria (5.9%) were the other metabolic abnormalities seen with lower prevalence levels. A total of 23% (n = 35) had both hypocitraturia and hypomagnesuria.

The distribution of these abnormalities varies between males and females, with some abnormalities being more prevalent in males than females. However, there was no significant association between gender and metabolic disorders (p = 0.061). There was a marginal association (p < 0.061) between age and metabolic disorders.

## Discussion

Age and sex have been shown to have an association with the prevalence of urolithiasis [8]. In our study, we observed a male predominance with a male-to-female ratio of 2:1 among patients treated for urolithiasis in our institution. The majority of these patients belonged to the age category of 40-59 years.

According to a study based on the Global Burden of Disease data, males had higher rates of global incidence for all age groups in 2019, with the highest rates occurring between ages 50 and 69 for both males and females [9]. A study analyzing the epidemiology of urolithiasis in Asia found a predisposition in Asian countries, with a male-to-female ratio ranging from 1.3 to 5. The study highlighted certain anatomical contrariety; the role of testosterone differences in certain dietary habits and climatic conditions are possible reasons for further exploration. The peak incidence of urolithiasis was observed in the age group of 30-60 years [8].

When looking at the Sri Lankan data, a study among 50 patients from different climatic zones found that the male-to-female ratio was 2.6:1 [10]. A study done in Northern Sri Lanka found that 74% of patients seeking surgical treatment for renal stones were males [11]. The majority of the patients belonged to the age group of 40-59. Overall, our findings on the sociodemographic epidemiology of urolithiasis resemble the findings of regional and national studies.

DM (23.5%) was the most common comorbidity among urolithiasis patients in this cohort, followed by hypertension, hyperlipidemia, and ischemic heart disease. This finding aligns with and supports the significance of insulin resistance in urinary tract stone formation. Nearly a quarter of patients (26.1%) had high serum creatinine at the time of the surgical procedure, which significantly improved in the majority following the unblocking of the renal system through surgical interventions. This underscores the importance of early intervention in urolithiasis patients with impaired renal function.

The prevalence of different types of stones based on chemical composition provides key insights into the major risk factors for stone disease in the population and the possible prevention strategies [12,13].

COM stones (78.4%) were by far the most prevalent stone type among our patients. UA stones, COD, cysteine, and CA stones were also found at lower percentages.

The prevalence of different types of stones varies significantly among countries and regions. In most countries in Asia, calcium oxalate (75-90%) is the most common component of stones, followed by UA (5-20%), calcium phosphate (6-13%), struvite (2-15%), apatite (1%), and cysteine (0.5-1%) [8]. A Chinese study among 507 patients from southern China found calcium oxalate (78.3%) to be the most prevalent stone type in China. UA stones were found in 3.6% and calcium phosphate in 3.4% of the patients [14].

According to an Indian study that analyzed 1,050 stones, 93.04% were calcium oxalate stones, out of which 80% were COM and 20% were COD. UA stones accounted for only 0.95% of the stones [15]. Our study aligns with the majority of the aforementioned findings from different studies, indicating that calcium oxalate stones are the most prevalent type, with calcium phosphate and cysteine constituting smaller percentages. However, our study observed a higher proportion of UA stones.

When looking at Sri Lankan studies that analyzed the chemical composition of stones in 50 patients from different climate zones of Sri Lanka, it was revealed that calcium oxalate stones constituted 86% of renal stones [8]. Phosphate and struvite calculi amounted to 2% each. Another study that analyzed the mineralogical and chemical composition of stones also found that the majority of urinary calculi were calcium oxalate stones. Struvite calculi, UA calculi, and hydroxyapatite calculi were the other prevailing types [16].

Focusing on Northern Sri Lanka, a study carried out among 104 patients with renal stones revealed that 75.9% of the patients had calcium oxalate and 13.5% had UA stones [17].

Our findings align with those of other Sri Lankan studies, highlighting consistency despite variations in climate and water quality. Notably, there was no significant regional difference in the prevalence of different types of stones, as observed by Kalana Hareendra et al. [10]. Interestingly, our study and a previous one conducted in the northern region revealed a higher proportion of UA stones compared to other Sri Lankan studies. It is noteworthy that the mean UA level among the patients was within the normal range, and only 6.5% of the patients exhibited hyperuricosuria.

The higher incidence of UA stones observed in our study may be attributed to various coexisting factors, including but not limited to a higher prevalence of insulin resistance [18], dietary habits [18], hereditary factors, and variations in drinking water quality. The higher prevalence of DM and the high acidic load resulting from rice consumption are the two most likely reasons that need to be explored. Further research is warranted in the future to comprehensively investigate and understand the interplay of these factors in the formation of kidney stones.

Low urine pH and low urine volume are two major etiologies for UA stones [19]. Since our findings did not reveal an increased prevalence of any hyperaciduric metabolic disorder among our patients, it is pertinent to assess the daily urine volumes of urolithiasis patients. This assessment might shed some light on the reasons for the higher prevalence of UA stones.

Urinary metabolic abnormalities are one of the most important risk factors for urinary stone disease and its recurrence. Urinary stasis, supersaturation of urine, and the formation of nidus are major pathologies that underlie urolithiasis [13]. Different metabolic factors either facilitate or inhibit the development of stone formation. Hypercalciuria, hyperoxaluria, hyperuricosuria, and hypercysteinuria are important metabolic derangements that promote urinary stone formation, while hypercitraturia and hypermagnesuria are deterrents [11,20,21].

Our study found that a large majority (86.9%) of the urolithiasis patients suffered from at least one urinary metabolic abnormality diagnosed by 24-hour urine analysis. The most common abnormality was hypocitraturia (60.1%), followed by hypomagnesuria (35.3%), hyperoxaluria (14.4%), hyperphosphaturia (11.1%), hyperuricosuria (6.5%), and hypercalciuria (5.9%).

Sri Lankan data on the prevalence of metabolic disorders among urolithiasis patients is largely lacking. A study done on 30 patients with recurrent stone disease in Northern Sri Lanka revealed that 80% of the patients suffered from a single metabolic derangement or a combination of multiple derangements [17]. Hyperoxaluria and hypomagnesuria were the most common metabolic disorders, with 30% of the patients suffering from each of the conditions. Hypocitraturia was found in only seven (23.3%) patients. A total of 10% of the patients had hyperuricosuria. Countrywide population studies are needed to find the actual prevalence of these metabolic disorders and understand the regional differences.

Limitation

This study was conducted at a specialized urology clinic providing tertiary care and involved 153 patients who underwent surgical treatment for urolithiasis. The approach may have introduced selection bias towards more severe cases, excluding those not requiring surgery, which could limit the applicability of the findings to settings with milder cases or conservative management. However, this study possessed several strengths, such as a comprehensive analysis of urolithiasis and detailed urinary metabolic evaluations for over 150 patients-a number that exceeds what is reported in the national literature. Due to financial constraints, serum analysis of electrolytes and metabolites could only be performed for 54 patients (all study samples underwent urine metabolic analysis), leading to partial profiles. To address these limitations, there is recognition of the necessity for longitudinal or cohort studies involving a wider range of urolithiasis patients, including those under conservative management or treated in outpatient settings, aiming to gain better insight into the progression of urolithiasis, identify risk factors, and assess intervention efficacy. Future research endeavors plan on utilizing longitudinal study designs to tackle these critical questions and provide stronger evidence for clinical decision-making.

## Conclusions

Our study elucidates significant insights into urolithiasis and its metabolic evaluation. Predominantly affecting middle-aged males, with ureteric colic as the primary symptom, our findings underscore the prevalence of recurrent stones and the predominance of COM and UA stones. Notably, metabolic abnormalities were prevalent among patients, with hypocitraturia and hypomagnesuria being the most common. While age showed no substantial association with stone type, a marginal correlation with metabolic disorders was observed. This highlights the intricate interplay between demographic factors, stone composition, and metabolic abnormalities in urolithiasis. Our study emphasizes the importance of tailored preventive strategies and personalized treatments to mitigate the burden of stone formation and recurrence effectively.
